# Microbial community succession in steam‐sterilized greenhouses infected with *Fusarium oxysporum*

**DOI:** 10.1111/1758-2229.13072

**Published:** 2022-04-20

**Authors:** Nilita Mukjang, Shorok B. Mombrikotb, Thomas Bell

**Affiliations:** ^1^ Department of Life Sciences Imperial College London Ascot Berkshire UK

## Abstract

*Fusarium* is an economically important crop pathogen but spends a large part of its life cycle in bulk soil environments where it interacts with a diverse community of soil microbes. Antagonistic interactions (e.g. competition) between the resident microbial community and *Fusarium* could constrain the growth of *Fusarium* in soil, which might therefore slow or prevent *Fusarium* establishment. We tracked *Fusarium oxysporum* in floriculture greenhouses where the soil had been steam‐sterilized to remove *Fusarium*. The data indicated a resurgence of soil bacteria and fungi during the first 90 days post‐sterilization, followed by a rapid decline in subsequent weeks, which was associated with an increase in *F*. *oxysporum* abundance at 148 days post sterilization. These changes over time were associated with successional changes in the bacterial but not the fungal communities. The results illustrate that, although soil steaming clears *Fusarium* in the short term, it may exacerbate re‐emergence as the resident community is continually depleted by the steaming process while *Fusarium* benefits from nutrients released by steaming. Observations suggest combining steaming with microbial inoculations could help reduce the recovery of *Fusarium* reducing the fungal load in the first instance and preventing subsequent build‐up by giving a head start to its saprophytic competitors.

## Introduction

Plant pathogens have had rising impacts on crops worldwide, with approximately 12% of total crop production lost to plant diseases (Reeleder, 2003). Emerging fungal infections are a prominent threat to food security, particularly to monoculture crops that are susceptible to new and virulent fungal lineages (Gurr *et al*., 2011). Consequently, there is great interest in tracking the dynamics of fungal pathogens in agro‐ecosystems, and in understanding the factors that control their spread.

Studies of fungal plant disease have largely focused on the molecular biology and epidemiology of plant–pathogen interactions, particularly on host defenses and pathogen virulence factors (Anderson *et al*., 2010). However, many fungal plant pathogens are soilborne, with substantial parts of their life history spent within the soil matrix rather than interacting directly with plants. Controlling soilborne pathogens is particularly challenging. Many biocontrol methods have been developed, but the main tool that has shown consistent results is to use plant breeding to select resistant plant cultivars. However, there are drawbacks to this approach. Selective plant breeding is time‐consuming and expensive, may only protect against a narrow range of pathogens (Panth *et al*., 2020; Zorrilla‐Fontanesi *et al*., 2020), and pathogens can evolve ways of bypassing new plant defenses (Burdon *et al*., 2016). Complementary approaches are therefore needed that can be used in addition to plant breeding and other biocontrol methods.

During growth and dispersal through the soil, fungal pathogens interact with other soil microbes, many of which can inhibit fungal growth through resource competition (Köhl *et al*., 2019) and direct interference, for example by secretion of inhibitory compounds or by otherwise modifying the environment (Garbeva *et al*., 2011; Schulz‐Bohm *et al*., 2017). These mechanisms have given rise to two explanations for the observation that the soil biota can suppress plant pathogens (Chandrashekara *et al*., 2012). First, ‘general suppression’ of plant pathogens in soil occurs due to diffuse competition for resources between the plant pathogen and the resident microbial community, comprised of hundreds of taxa competing for soil resources (Weller *et al*., 2002; Schlatter *et al*., 2017). Second, ‘specific suppression’ in soil occurs due to individual antagonisms between identifiable taxa and the plant pathogen, for example due to the secretion of a specific inhibitory chemical (Expósito *et al*., 2017).

These ideas are consistent with invasion ecology theory, where the degree to which resident communities resist new invaders is determined by the composition and diversity of the resident community (Vila *et al*., 2019). Communities with different compositions are likely to contain taxa that have differing degrees of niche overlap with the invasive species. Similarly, more diverse communities are likely to have more tightly packed niches, leaving little available niche space for the invader (Eisenhauer *et al*., 2013), and are also more likely to contain taxa that secrete substrates that are inhibitory to the invader. Consistent with these ideas, a long‐term grassland experiment showed the highest disease suppression of *Rhizoctonia solani* AG3 was found in plots with highest soil microbial diversity (Brussaard *et al*., 2007). Prior studies have also shown that pathogen suppression is increased in fields with higher microbial soil diversity (Reeleder, 2003). Experimental studies have also shown that perturbations to the resident soil community result in a loss of pathogen suppression and an increase in pathogen abundance (Mendes *et al*., 2011).

Therefore, there is a need to track microbial dynamics of bulk soil during pathogen ‘invasions’ to understand the role of resident communities in pathogen suppression. This might be particularly important when the soil microbial communities are perturbed because perturbations provide a window of opportunity for pathogen invasions. We therefore looked at how changes in soil microbial communities were correlated with the ability of the plant pathogen *Fusarium oxysporum* (Booth, 1971) to invade commercial greenhouses that had received strong soils perturbations.

*Fusarium oxysporum* is a genetically heterogeneous species complex (Dongzhen *et al*., 2020) that causes enormous economic damage to a wide range of crops worldwide (DEAN *et al*., 2012). *Fusarium oxysporum* is a cosmopolitan soil inhabitant, with genetic variants that are both free‐living and opportunistic pathogens across a wide variety of crops. It exists as spores or mycelia in bulk soil (Gordon, 2017) and needs to be actively or passively dispersed to the plant surface to infect plants (Dita *et al*., 2018). Soil sterilization using steam is one of the methods used to mitigate *Fusarium*. The efficiency of the steam and the target organism is temperature‐dependent. At ~50°C, nematodes, some oomycetes and other water moulds are killed (Abbas, 2015). Most plant pathogenic fungi and bacteria, some worms, slugs and centipedes are usually killed between 60°C and 72°C. At 82°C, the heat can kill most weeds, the rest of the plant pathogenic bacteria, most plant viruses in plant debris, and most insects (Abbas, 2015). Heat‐tolerant weed seeds and some plant viruses, such as tobacco mosaic virus are killed at or near the boiling point (between 95°C and 100°C) (Abbas, 2015). However, with this method, *Fusarium* can still re‐emerge in the soil.

Here, we surveyed floricultural greenhouses of scented stocks (*Matthiola incana*), which are being infected by an emerging and recurring *Fusarium* wilt disease (Baker, 1948), identified as *F*. *oxysporum f*. sp. *mathioli* (Green and O'Neill, 2007). *Fusarium oxysporum f*. sp. *mathioli* causes vascular wilt disease, with symptoms including softening of leaves and stem collapse, which can result in widespread mortality before harvest. Diseased plants also exhibit stunted growth, bleaching on the leaves or green leaves with yellow veins (leaf netting effect), and dark brown vascular staining within the stem, which can also have significant financial repercussions for growers (O'Neill and Green, 2010). This study asks how agricultural pathogens are spread and maintained in floricultural site. Currently, growers use steam to sterilize the soil to suppress *F*. *oxysporum*. They also focus on flower species with short turnovers to limit the exposure time. We surveyed bulk soil from four greenhouses and profiled the bacterial and fungal communities while quantifying the *F*. *oxysporum* load. To assess the efficiency of soil steaming as a mitigation method, we sampled multiple times, points from steaming to harvest, while tracking soil *Fusarium*. We hypothesised that there were reservoirs of *Fusarium* deeper in the soil where steam did not penetrate (~20 cm). We therefore surveyed both *Fusarium* and soil bacterial and fungal communities at different depths to quantify the *Fusarium* load and to search for correlations with microbial community changes.

## Results and discussion

### *Bacterial, fungal and* Fusarium *dynamics in greenhouse soils*

**Fig. 1:**
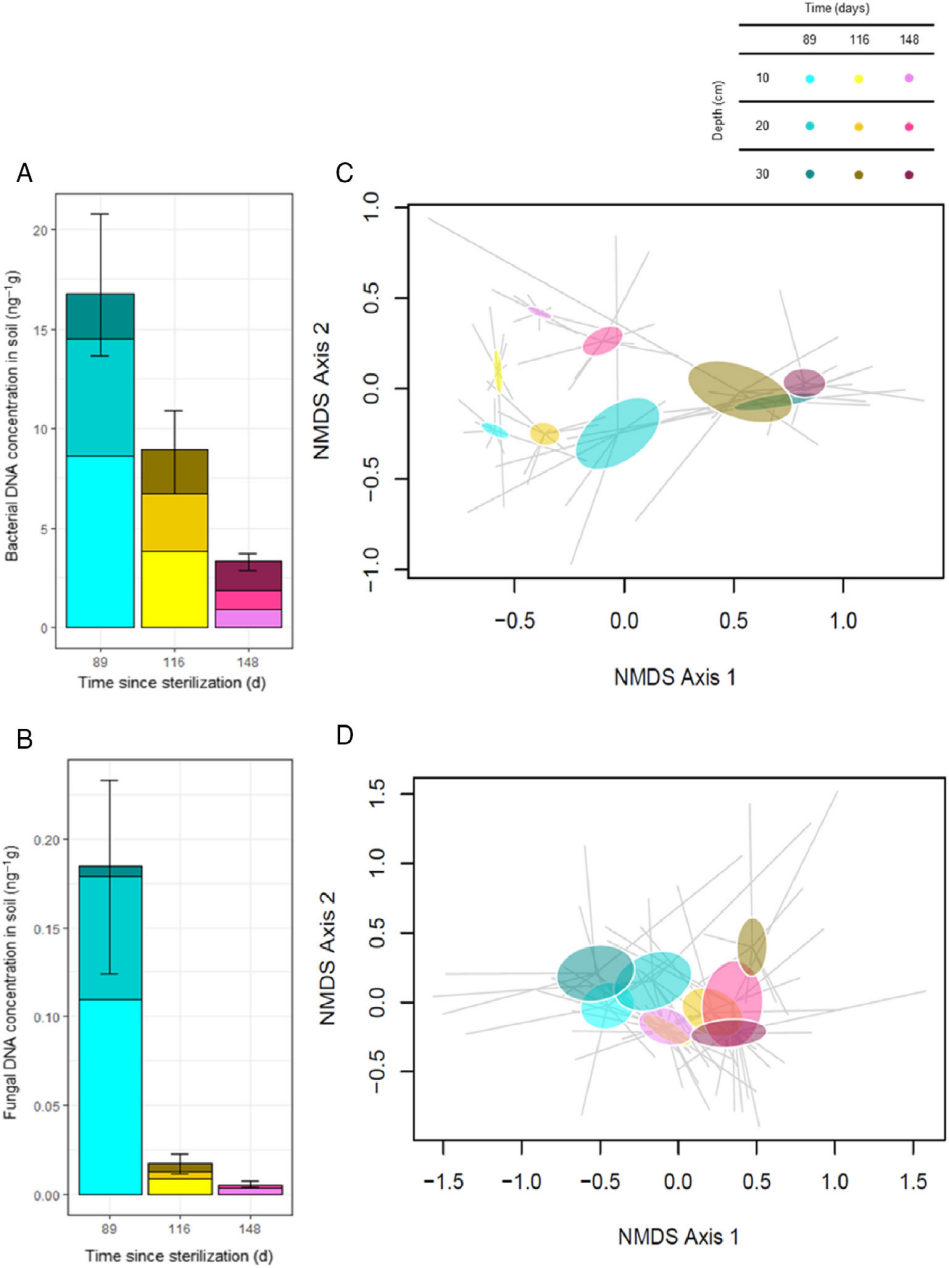
Bacterial (A) and fungal (B) DNA concentrations in soil (ng g^−1^) and non‐metric multidimensional scaling analysis of bacterial (C; Stress = 0.132) and fungal (D; Stress = 0.232) communities over time post soil steam sterilization at three depths (0–10, 10–20 and 20–30 cm). Soil samples are from four greenhouses (White Gate) at Tuxhill Farm.

**Fig. 2:**
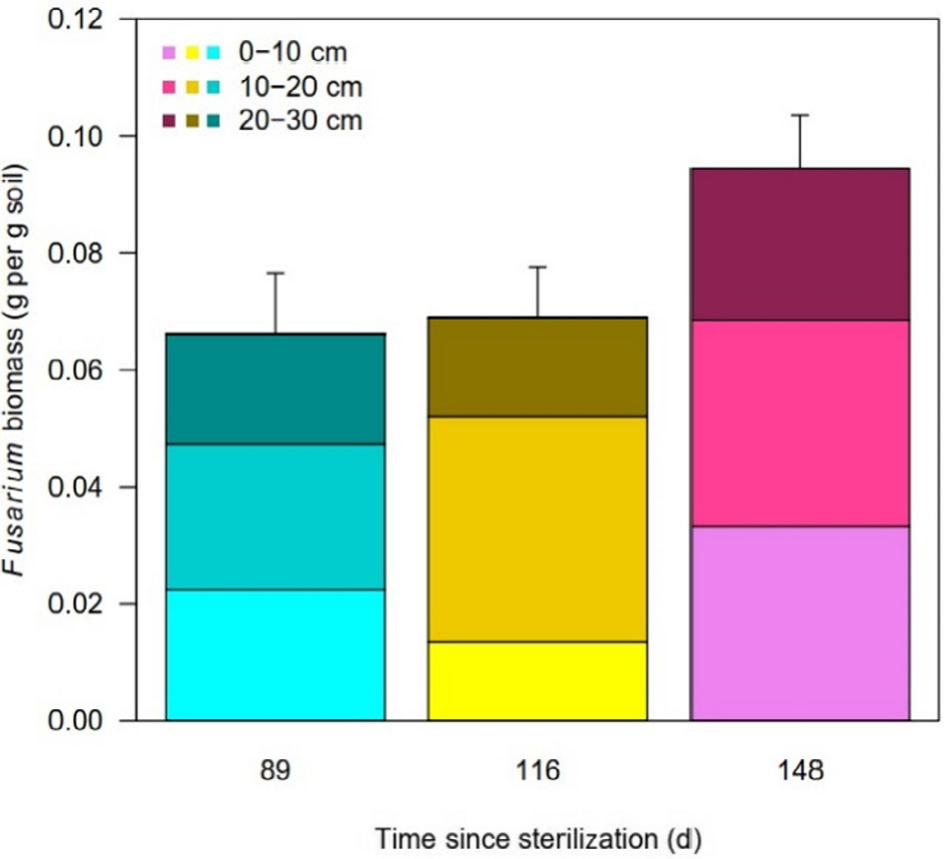
Average *Fusarium* biomass (g) per gram of soil after incubated at room temperature in MGB for 7 days. Broth was inoculated with soil washes from soil samples taken from different greenhouses at Tuxhill Farm at multiple times points post soil steam sterilization at three depths from the top soil (0–10, 10–20 and 20–30 cm).

Soil bacterial and fungal communities declined in abundance over the study period (Fig. 1A and B). Across all greenhouses, the first sampling data (89 days post‐sterilization) had the highest bacterial and fungal abundance across all four greenhouses. Abundances declined precipitously in subsequent sampling days. Overall, there was a significant decline in bacterial (lme: *df* = 61, *p* ≤ 0.0001) and fungal abundance (lme: *df* = 58, *p* = 0.0001) in all of the greenhouses, but no difference in abundance with greenhouses. At the same time, *Fusarium* biomass was stable from Day 89 to Day 116 but subsequently increased on Day 148 (lme: *df* = 67, *p* = 0.0327) (Fig. 2).

The decline in bacterial abundance was associated with substantial changes in bacterial community composition. Ordination of the bacterial communities showed that there were systematic changes over time throughout the soil profile (left to right on NMDS axis 1) (Fig. 1C). Communities were distinct across the three depths in the first and second sampling dates but appeared to homogenize at the final time point. Reflecting this observation, bacterial community composition was impacted by time (PERMANOVA: *F* = 4.734 with *df* = 2, *p* = 0.002), depth (PERMANOVA: *F* = 19.345 with *df* = 2, *p* = 0.001) and the interaction between time and depth (PERMANOVA: *F* = 1.384 with *df* = 4, *p* = 0.112). Similarly, fungal community composition (Fig. 1D) was impacted by time (PERMANOVA: *F* = 3.204 with *df* = 2, *p* = 0.003), depth (PERMANOVA: *F* = 2.200 with *df* = 2, *p* = 0.012) and the interaction between time and depth (PERMANOVA: *F* = 0.862 with *df* = 4, *p* = 0.645). The OTU richness increased with soil depth for both the bacteria and the fungi (ANOVA: bacteria: *F*_2,48_ = 6.73, *p* = 0.0026, fungi: *F*_2,48_ = 5.94, *p* = 0.0054), but there was no impact of time‐since‐sterilization on bacterial or fungal OTU richness (no significant main effect or interaction terms).

Successional changes in bacterial community composition were associated with an increase in the abundance of *F*. *oxysporum* in greenhouse soil by the final sampling date. While this result is correlative, it does imply that the bulk soil microbial communities play a role in *F*. *oxysporum* dynamics. The finding is broadly aligned with studies that have shown that at least some plant pathogen suppression can be attributed to the composition of the soil microbial communities (Ou *et al*., 2019). The large turnover in bacterial community composition that we observed indicates swiftly changing conditions in the greenhouse soil post‐steaming, some of which would allow greater access to invading pathogenic spores and mycelia. The much higher bacterial and fungal abundances in the weeks immediately after steaming imply a substantial release of nutrients due to steaming. Others have found that sterilization does indeed release a pulse of nutrients, particularly phosphorus, resulting in short‐lived increases in microbial abundance (Dietrich *et al*., 2020). While this would allow rapid re‐colonization by the resident microbial community, it would also create a window of opportunity for *Fusarium* to re‐establish when nutrients are plentiful.

## Conclusion

These observations raise questions about the shifting role of microbial composition in suppressing plant pathogens, and the efficacy of soil steaming as a method of pathogen mitigation. The observations imply a stronger role of ‘specific’ suppression soon after steaming, when high microbial abundances also provide the potential for high production of inhibitory compounds by specific antagonists. By contrast, we predict ‘general’ suppression becomes more important over time after steaming as nutrients become limiting for growth and as microbial abundances and soil nutrients decline as a consequence. The observations also raise the spectre that, although soil steaming clears *Fusarium* over the short term, it may exacerbate future bouts of *Fusarium* infection since the resident community is continually being serially depleted by the steaming process while also ‘feeding’ *Fusarium* with nutrients released by steaming. A combination of steaming and microbial inoculations or transplants could be an effective way to reduce the recovery of *Fusarium* by not just clearing *Fusarium* from soil but also preventing its subsequent build‐up by giving a head start to its saprophytic competitors.

## Experimental procedures

### Fusarium oxysporum *dynamics in floriculture greenhouses*

Soil samples were taken from four commercial greenhouses (White Gate; mean greenhouse size 2261 m^2^) at Tuxhill Farm located in Norfolk (United Kingdom). All four greenhouses have had recurring bouts of *F*. *oxysporum* infection that have impacted scented stock (*Matthiola incana*), reducing flower quantity by approximately 5% and in some instances causing failure of whole harvests. To avoid chemical intervention, the farm uses steam to sterilize the top ~30 cm of soil. However, *F*. *oxysporum* infection re‐occurs every growing season, so repeated steaming is required to prepare the soil before planting new crops, resulting in increased labor and fuel costs.

Within each greenhouse, the top 20 cm of surface soil is rotated annually and sterilized by steaming prior to planting with seedlings. Steaming was conducted by covering 14 m × 20 m with thermally stable plastic sheets secured around the edges. The steam leaves the boiler as superheated dry steam at 230°C and was pumped under the sheet. After fully inflating the steaming sheet, steaming was continuing for 7 h. Soil was left for 3–4 days after which 1‐month‐old seedlings were planted. Plants were watered two to three times a week. Data from the four greenhouses were selected because they were steamed on the same day, allowing for a replicated longitudinal study of the impacts of steaming on the soil communities and on *Fusarium* abundances.

### Greenhouse soil sampling

Bulk soil was sampled from the greenhouses using a 60 cm‐long soil core with 1.2 cm diameter. Soil samples were taken at the first row of water pipeline in each greenhouse. Five soil cores were taken at 2, 8, 14, 20 and 24 m from the central walk path. The corer was sterilized using ethanol between each sample. Each soil core was separated into three depths: 0–10, 10–20 and 20–30 cm. We pooled the soil across the five samples at each depth, and the pooled soil was homogenized by hand in a sterile bag. Homogenized soil samples were subsampled, adding approximately 0.5 g into a 2 ml bashing tube containing 750 μl of Genomic lysis buffer (Zymo Research). Samples were lysed on site using handheld bead beater (Zymo Terralyser). The exact soil weight was determined prior to DNA extraction by using the pre and post lysis tube weight. The lysis tubes were kept at room temperature until returning to the lab, upon which they were frozen at −80°C until the day of DNA extraction. The remaining bulk soil samples were kept cold at approximately 4°C until returning to the lab where they were refrigerated at 4°C.

### *Greenhouse* F*.* oxysporum *biomass*

Biomass assays were used to quantify live *Fusarium* present in the soil. Homogenized soil samples were subsampled into 15 ml centrifuge tubes. Soil in the tubes was weighed and phosphate buffer saline (Sigma) was added in 1:1 (ml g^−1^) of soil to get soil wash. The samples were vortexed for 5 s to allow microorganism within soil matrix to be extracted and stored in an aqueous state. Samples were mixed using an orbital shake (Stuart SSL1) for 10 min at 225 rpm and placed at room temperature for 10 min to allow soil to settle out. 500 μl of soil wash was added to 10 ml Malachite green broth (MGB) (Castellá *et al*., 1997) in 50 ml centrifuge tubes. The tubes were incubated horizontally at room temperature for 7 days then centrifuged at 6000 rpm, 25°C for 10 min. The supernatant was discarded and the cells were washed twice with 10 ml of deionized water. The samples were dried at 70°C, after which they were weighted to calculate *Fusarium* biomass by subtracting the known weight of the centrifuge tube.

### Bacterial and fungal abundance

Soil samples from the greenhouses (approximately 0.25 g) were taken for DNA extraction using the Quick‐DNA™ Faecal/Soil Microbe 96 Kit (Zymo Research) and following the manufacturer's instructions. Lysis tubes were thawed at room temperature (21°C) and samples were lysed again for 1 min using a handheld bead beater. Extracted DNA was used to quantify bacterial and fungal abundances using qPCR and for community analysis using amplicon sequencing.

We used qPCR to quantify bacteria and fungi in the soil. We used genomic DNA from *Escherichia coli* K‐12 (for bacteria; 16S) and *Aspergillus fumigatus* (for fungi; ITS) as standards for generating calibration curves. Standards were quantified using Quant‐iT dsDNA HS Assay Kit (Invitrogen Life Technologies), and four 10‐fold dilutions were used to generate a standard curve for each qPCR plate. Universal bacterial and fungal primers were used; 515F/806R bacterial and ITS7/ITS4 for fungi. Each 25.0 μl reaction consisted of: forward primer 1.25 μl, reverse primer 1.25 μl, SYBR green mixture 12.5 μl, DNA 5.0 μl, Nucleases free H_2_O 5.0 μl. Cycling conditions for 16S rRNA gene (bacteria) were: initial denaturation at 95°C for 15 min followed by 40 cycles of denaturation at 95°C for 45 s, annealing at 50°C for 60 s, extension at 72°C for 90 s, and a final extension at 72°C for 10 min. Cycling conditions for ITS gene (fungi) were: initial denaturation at 95°C for 15 min, followed by 40 cycles of denaturation at 95°C for 30 s, annealing at 56°C for 30 s, extension at 68°C for 30 s, and a final extension at 72°C for 10 min. The cycle was completed with a melt curve consisting of 95°C, 10 s/65°C, 60 s/97°C, 1 s. All qPCRs were conducted on a LightCycler® 96 SW 1.1 instrument (Roche). qPCR copy number was calculated from the standard curves and are reported as copy numbers per gram of soil.

### Amplicon sequencing of laboratory and greenhouse microbial communities

We used two‐step PCR for amplicon sequencing of the bacterial and fungal communities. In the first step, each 50.0 µl reaction consisted of: 100 µM forward primer (Bacteria:16S 515F nexteraV1; Fungi: ITS7 F nextera, Appendix Table A1‐A3) 0.1 µl, 100 µM reverse primer (Bacteria:16S 806R nexteraV1; Fungi: ITS4 R nextera, Appendix Table A1‐A3) 0.1 µl, Taq DNA Polymerase (Sigma‐Aldrich) 0.25 µl, 10X PCR buffer 5.0 µl, Bovine serum albumin (Sigma‐Aldrich) 0.5 µl, 10 mM dNTP Blend (GeneAmp) 1.0 µl, DNA 5.0 µl, nucleases free H2O 38.05 µl. The cycling conditions for 16S rRNA gene (bacteria) were: initial denaturation at 94°C for 3 min followed by 35 cycles of denaturation at 94°C for 45 s, annealing at 50°C for 60 s, extension at 72°C for 90 s repeating, and a final extension at 72°C for 10 min. The cycling conditions for ITS gene (fungi) were: initial denaturation at 95°C for 3 min, followed by 40 cycles of denaturation at 95°C for 15 s, annealing at 52°C for 30 s, extension at 72°C for 30 s, and a final extension at 72°C for 10 min. In the second step, for both 16S rRNA gene and ITS gene, whereby the unique barcodes and adaptors are attached to the first step amplicons (Appendix Table A1‐A3) were: each 50.0 µl PCR reaction for bacteria and fungi consisted of: 1.25 µM index forward primer 5.0 µl, 1.25 µM index reverse primer 5.0 µl, Taq DNA Polymerase (Sigma‐Aldrich) 0.25 µl, 10X PCR buffer 5.0 µl, 10 mM dNTP Blend (GeneAmp) 1.0 µl, DNA 5.0 µl (~20 ng), nucleases free H2O 28.75 µl. The cycling conditions were: initial denaturation at 95°C for 2 min, followed by 8 cycles of denaturation at 95°C for 15 s, annealing at 55°C for 30 s, extension at 72°C for 30 s, and a final extension at 72°C for 10 min. Amplicon libraries were then sequenced on the MiSeq platform (© 2018 Illumina).

Amplicon reads generated by the MiSeq sequencing were processed using the *DADA2* pipeline (Callahan *et al*., 2016) in *R*. We used the *phyloseq* package in *R* to generate OTU tables which were used for downstream analysis. Bacterial and fungal communities were visualized using non‐metric dimensional scaling using the metaMDS function in the *vegan* package. We used permutational analysis of variance (adonis function in *vegan*) to identify whether compositional differences related to *F*. *oxysporum* abundance. The linear mixed‐effects models were employed to identify whether time since sterilization impacted soil bacterial and fungal communities declined in abundance. We calculated the diversity (OTU richness) of each community, and used analysis of variance to quantify the impact of soil depth and time since sterilization on diversity.

## Supporting information


**Fig. S1.** Phylum‐level of bacterial composition (A) and class‐level fungal composition (B) in White Gate greenhouse (WG) through time since last soil sterilization (steaming) at depth 0–30 cm from the topsoil.
**Fig. S2.** Bacterial and fungal DNA concentrations in the PH7 greenhouse where soil was steamed for a day.

**Table A1.** The sequences of primers for bacterial and fungal communities sequencing for 1^st^ step PCR.
**Table A2.** The sequences of primer for bacterial and fungal communities sequencing for 2^nd^ step PCR.
**Table A3.** Primer array combinations for bacterial and fungal communities sequencing for 2^nd^ step PCR.

## References

[emi413072-bib-0001] Abbas, A. (2015) . In Management of Plant Diseases, 1st ed. Abbas, A. (ed). Peshawar, Pakistan: The University of Agriculture.

[emi413072-bib-0002] Anderson, J.P. , Gleason, C.A. , Foley, R.C. , Thrall, P.H. , Burdon, J.B. , and Singh, K.B. (2010) Plants versus pathogens: an evolutionary arms race. Funct Plant Biol 37: 499–512. 21743794 10.1071/FP09304 PMC3131095

[emi413072-bib-0003] Baker, K. (1948) *Fusarium* wilt of garden stock (*Mathiola incana*). Phytopathology 38: 399–403.

[emi413072-bib-0004] Booth, C. (1971) The Genus Fusarium. Kew, UK: Commonwealth Mycological Institute.

[emi413072-bib-0005] Brussaard, L. , de Ruiter, P.C. , and Brown, G.G. (2007) Soil biodiversity for agricultural sustainability. Agric Ecosyst Environ 121: 233–244.

[emi413072-bib-0006] Burdon, J.J. , Zhan, J. , Barrett, L.G. , Papaix, J. , and Thrall, P.H. (2016) Addressing the challenges of pathogen evolution on the world's arable crops. Phytopathology 106: 1117–1127. 27584868 10.1094/PHYTO-01-16-0036-FI

[emi413072-bib-0007] Callahan, B.J. , McMurdie, P.J. , Rosen, M.J. , Han, A.W. , Johnson, A.J.A. , and Holmes, S.P. (2016) DADA2: high‐resolution sample inference from Illumina amplicon data. Nat Methods 13: 581–583. 27214047 10.1038/nmeth.3869 PMC4927377

[emi413072-bib-0008] Castellá, G. , Bragulat, M.R. , Rubiales, M.V. , and Cabañes, F.J. (1997) Malachite Green Agar, a New Selective Medium for Fusarium spp. Netherlands: Kluwer Academic Publishers. 10.1023/A:1006886529511 16283458

[emi413072-bib-0009] Chandrashekara, C. , Kumar, R. , Bhatt, J.C. , and Chandrashekara, K.N. (2012) Suppressive soils in plant disease management. In Eco‐friendly Innovative Approaches in Plant Disease Management. India: International Book Distributors, pp. 241–256.

[emi413072-bib-0010] Dean, R. , Van Kan, J.A.L. , Pretorius, Z.A. , Hammond‐Kosack, K.E. , Di Pietro, A. , Spanu, P.D. , *et al*. (2012) The top 10 fungal pathogens in molecular plant pathology. Mol Plant Pathol 13: 414–430. 22471698 10.1111/j.1364-3703.2011.00783.x PMC6638784

[emi413072-bib-0011] Dietrich, P. , Cesarz, S. , Eisenhauer, N. , and Roscher, C. (2020) Effects of steam sterilization on soil abiotic and biotic properties. Soil Org 92: 99–108.

[emi413072-bib-0012] Dita, M. , Barquero, M. , Heck, D. , Mizubuti, E.S.G. , and Staver, C.P. (2018) Fusarium wilt of banana: current knowledge on epidemiology and research needs toward sustainable disease management. Front Plant Sci 871: 1–21. 10.3389/fpls.2018.01468 PMC6202804 30405651

[emi413072-bib-0013] Dongzhen, F. , Xilin, L. , Xiaorong, C. , Wenwu, Y. , Yunlu, H. , and Yi, C. (2020) Fusarium species and Fusarium oxysporum species complex genotypes associated with yam wilt in South‐Central China. Front Microbiol 11: 1–17. 33013737 10.3389/fmicb.2020.01964 PMC7461894

[emi413072-bib-0014] Eisenhauer, N. , Schulz, W. , Scheu, S. , and Jousset, A. (2013) Niche dimensionality links biodiversity and invasibility of microbial communities. Funct Ecol 27: 282–288.

[emi413072-bib-0015] Expósito, R.G. , de Bruijn, I. , Postma, J. , and Raaijmakers, J.M. (2017) Current insights into the role of rhizosphere bacteria in disease suppressive soils. Front Microbiol 8: 1–12. 29326674 10.3389/fmicb.2017.02529 PMC5741648

[emi413072-bib-0016] Garbeva, P. , Hol, W.H.G. , Termorshuizen, A.J. , Kowalchuk, G.A. , and De Boer, W. (2011) Fungistasis and general soil biostasis – a new synthesis. Soil Biol Biochem 43: 469–477.

[emi413072-bib-0017] Gordon, T.R. (2017) Fusarium oxysporum and the fusarium wilt syndrome. Annu Rev Phytopathol 55: 23–39. 28489498 10.1146/annurev-phyto-080615-095919

[emi413072-bib-0018] Green, K. , and O'Neill, T. (2007) Integrated management of stock fusarium wilt. Hortic Dev Counc. https://projectblue.blob.core.windows.net/media/Default/Horticulture/Diseases/stock%20fusarium.pdf

[emi413072-bib-0019] Gurr, S. , Samalova, M. , and Fisher, M. (2011) The rise and rise of emerging infectious fungi challenges food security and ecosystem health. *Fungal* . Biol Rev 25: 181–188.

[emi413072-bib-0020] Köhl, J. , Kolnaar, R. , and Ravensberg, W.J. (2019) Mode of action of microbial biological control agents against plant diseases: relevance beyond efficacy. Front Plant Sci 10: 845. 31379891 10.3389/fpls.2019.00845 PMC6658832

[emi413072-bib-0021] Mendes, R. , Kruijt, M. , De Bruijn, I. , Dekkers, E. , Van Der Voort, M. , Schneider, J.H.M. , *et al*. (2011) Deciphering the rhizosphere microbiome for disease‐suppressive bacteria. Science 332: 1097–1100. 21551032 10.1126/science.1203980

[emi413072-bib-0022] O'Neill, T.M. , and Green, K.R. (2010) Evaluation of some pre‐plant soil treatments and chemical disinfectants for control of fusarium wilt diseases in protected cut flowers. Acta Hortic 883: 215–222.

[emi413072-bib-0023] Ou, Y. , Penton, C.R. , Geisen, S. , Shen, Z. , Sun, Y. , Lv, N. , *et al*. (2019) Deciphering underlying drivers of disease suppressiveness against pathogenic fusarium oxysporum. Front Microbiol 10: 1–12. 31781059 10.3389/fmicb.2019.02535 PMC6861331

[emi413072-bib-0024] Panth, M. , Hassler, S.C. , and Baysal‐Gurel, F. (2020) Methods for management of soilborne diseases in crop production. Agriculture 10: 1–21.

[emi413072-bib-0025] Reeleder, R.D. (2003) Fungal plant pathogens and soil biodiversity. Can J Soil Sci 83: 331–336.

[emi413072-bib-0026] Schlatter, D. , Kinkel, L. , Thomashow, L. , Weller, D. , and Paulitz, T. (2017) Disease suppressive soils: new insights from the soil microbiome. Phytopathology 107: 1284–1297. 28650266 10.1094/PHYTO-03-17-0111-RVW

[emi413072-bib-0027] Schulz‐Bohm, K. , Martín‐Sánchez, L. , and Garbeva, P. (2017) Microbial volatiles: small molecules with an important role in intra‐ and inter‐kingdom interactions. Front Microbiol 8: 1–10. 29312193 10.3389/fmicb.2017.02484 PMC5733050

[emi413072-bib-0028] Vila, J.C.C. , Jones, M.L. , Patel, M. , Bell, T. , and Rosindell, J. (2019) Uncovering the rules of microbial community invasions. Nat Ecol Evol 3: 1162–1171. 31358951 10.1038/s41559-019-0952-9

[emi413072-bib-0029] Weller, D.M. , Raaijmakers, J.M. , McSpadden Gardener, B.B. , and Thomashow, L.S. (2002) Microbial populations responsible for specific soil suppressiveness to plant pathogens. Annu Rev Phytopathol 40: 309–348. 12147763 10.1146/annurev.phyto.40.030402.110010

[emi413072-bib-0030] Zorrilla‐Fontanesi, Y. , Pauwels, L. , Panis, B. , Signorelli, S. , Vanderschuren, H. , and Swennen, R. (2020) Strategies to revise agrosystems and breeding to control fusarium wilt of banana. Nat Food 1: 599–604. 10.1038/s43016-020-00155-y 37128105

