# Supplementary material for: Microbial community succession in steam‐sterilized greenhouses infected with Fusarium oxysporum
Source: Environ Microbiol Rep. 2022 Apr 20;14(4):577–83. doi: 10.1111/1758-2229.13072 (PMC9544407; doi:10.1111/1758-2229.13072)
Supplement: Supplementary file 1 — Fig. S1. Phylum‐level of bacterial composition (A) and class‐level fungal composition (B) in White Gate greenhouse (WG) through time since last soil sterilization (steaming) at depth 0–30 cm from the topsoil. Fig. S2. Bacterial and fungal DNA concentrations in the PH7 greenhouse where soil was steamed for a day. [file EMI4-14-577-s001.docx]

**Supplementary**

(A)

**
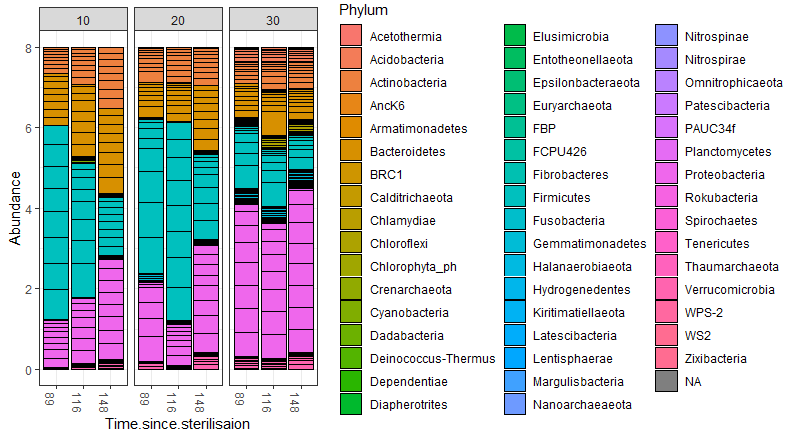
**
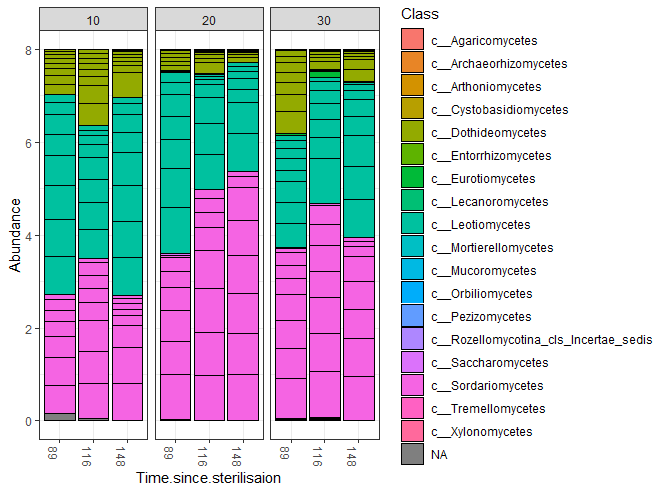


(B)

**Figure S1** Phylum-level of bacterial composition (A) and class-level fungal composition (B) in White gate greenhouse (WG) through time since last soil sterilisation (steaming) at depth 0-30 cm from the topsoil.


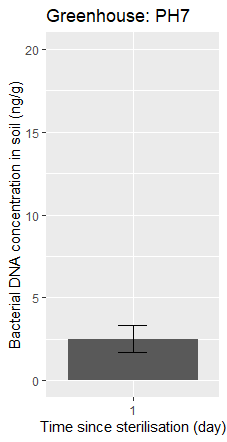

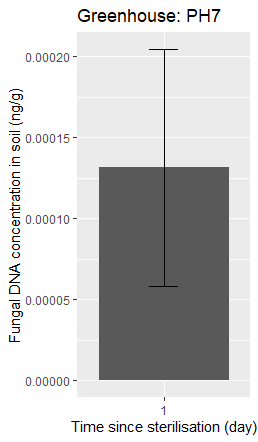


**Figure S2** Bacterial and fungal DNA concentrations in the PH7 greenhouse where soil was steamed for a day.
