# Supplementary material for: Microbial community succession in steam‐sterilized greenhouses infected with Fusarium oxysporum
Source: Environ Microbiol Rep. 2022 Apr 20;14(4):577–83. doi: 10.1111/1758-2229.13072 (PMC9544407; doi:10.1111/1758-2229.13072)
Supplement: Supplementary file 2 — Table A1. The sequences of primers for bacterial and fungal communities sequencing for 1st step PCR. Table A2. The sequences of primer for bacterial and fungal communities sequencing for 2nd step PCR. Table A3. Primer array combinations for bacterial and fungal communities sequencing for 2nd step PCR. [file EMI4-14-577-s002.docx]

## **Appendix**

**Table A1.** The sequences of primers for bacterial and fungal communities sequencing for 1^st^ step PCR.

| **Primer** | **Sequence** |
| --- | --- |
| 16S_515F_nexteraV1 | TCGTCGGCAGCGTCAGATGTGTATAAGAGACAGGTGYCAGCMGCCGCGGTAA |
| 16S_806R_nexteraV1 | GTCTCGTGGGCTCGGAGATGTGTATAAGAGACAGGGACTACNVGGGTWTCTAAT |
| ITS7_F_nextera | TCGTCGGCAGCGTCAGATGTGTATAAGAGACAGGTGARTCATCGAATCTTTG |
| ITS4_R_nextera | GTCTCGTGGGCTCGGAGATGTGTATAAGAGACAGTCCTCCGCTTATTGATATGC |

**Table A2.** The sequences of primer for bacterial and fungal communities sequencing for 2^nd^ step PCR.

| **Primer** | **Sequence** |
| --- | --- |
| SA501 | AATGATACGGCGACCACCGAGATCTACACATCGTACGTCGTCGGCAGCGTC |
| SA502 | AATGATACGGCGACCACCGAGATCTACACACTATCTGTCGTCGGCAGCGTC |
| SA503 | AATGATACGGCGACCACCGAGATCTACACTAGCGAGTTCGTCGGCAGCGTC |
| SA504 | AATGATACGGCGACCACCGAGATCTACACCTGCGTGTTCGTCGGCAGCGTC |
| SA505 | AATGATACGGCGACCACCGAGATCTACACTCATCGAGTCGTCGGCAGCGTC |
| SA506 | AATGATACGGCGACCACCGAGATCTACACCGTGAGTGTCGTCGGCAGCGTC |
| SA507 | AATGATACGGCGACCACCGAGATCTACACGGATATCTTCGTCGGCAGCGTC |
| SA508 | AATGATACGGCGACCACCGAGATCTACACGACACCGTTCGTCGGCAGCGTC |
| SB501 | AATGATACGGCGACCACCGAGATCTACACCTACTATATCGTCGGCAGCGTC |
| SB502 | AATGATACGGCGACCACCGAGATCTACACCGTTACTATCGTCGGCAGCGTC |
| SB503 | AATGATACGGCGACCACCGAGATCTACACAGAGTCACTCGTCGGCAGCGTC |
| SB504 | AATGATACGGCGACCACCGAGATCTACACTACGAGACTCGTCGGCAGCGTC |
| SB505 | AATGATACGGCGACCACCGAGATCTACACACGTCTCGTCGTCGGCAGCGTC |
| SB506 | AATGATACGGCGACCACCGAGATCTACACTCGACGAGTCGTCGGCAGCGTC |
| SB507 | AATGATACGGCGACCACCGAGATCTACACGATCGTGTTCGTCGGCAGCGTC |
| SB508 | AATGATACGGCGACCACCGAGATCTACACGTCAGATATCGTCGGCAGCGTC |
| SA701 | CAAGCAGAAGACGGCATACGAGATAACTCTCGGTCTCGTGGGCTCGG |
| SA702 | CAAGCAGAAGACGGCATACGAGATACTATGTCGTCTCGTGGGCTCGG |
| SA703 | CAAGCAGAAGACGGCATACGAGATAGTAGCGTGTCTCGTGGGCTCGG |
| SA704 | CAAGCAGAAGACGGCATACGAGATCAGTGAGTGTCTCGTGGGCTCGG |
| SA705 | CAAGCAGAAGACGGCATACGAGATCGTACTCAGTCTCGTGGGCTCGG |
| SA706 | CAAGCAGAAGACGGCATACGAGATCTACGCAGGTCTCGTGGGCTCGG |
| SA707 | CAAGCAGAAGACGGCATACGAGATGGAGACTAGTCTCGTGGGCTCGG |
| SA708 | CAAGCAGAAGACGGCATACGAGATGTCGCTCGGTCTCGTGGGCTCGG |
| SA709 | CAAGCAGAAGACGGCATACGAGATGTCGTAGTGTCTCGTGGGCTCGG |
| SA710 | CAAGCAGAAGACGGCATACGAGATTAGCAGACGTCTCGTGGGCTCGG |
| SA711 | CAAGCAGAAGACGGCATACGAGATTCATAGACGTCTCGTGGGCTCGG |
| SA712 | CAAGCAGAAGACGGCATACGAGATTCGCTATAGTCTCGTGGGCTCGG |
| SB701 | CAAGCAGAAGACGGCATACGAGATAAGTCGAGGTCTCGTGGGCTCGG |
| SB702 | CAAGCAGAAGACGGCATACGAGATATACTTCGGTCTCGTGGGCTCGG |
| SB703 | CAAGCAGAAGACGGCATACGAGATAGCTGCTAGTCTCGTGGGCTCGG |
| SB704 | CAAGCAGAAGACGGCATACGAGATCATAGAGAGTCTCGTGGGCTCGG |
| SB705 | CAAGCAGAAGACGGCATACGAGATCGTAGATCGTCTCGTGGGCTCGG |
| SB706 | CAAGCAGAAGACGGCATACGAGATCTCGTTACGTCTCGTGGGCTCGG |
| SB707 | CAAGCAGAAGACGGCATACGAGATGCGCACGTGTCTCGTGGGCTCGG |
| SB708 | CAAGCAGAAGACGGCATACGAGATGGTACTATGTCTCGTGGGCTCGG |
| SB709 | CAAGCAGAAGACGGCATACGAGATGTATACGCGTCTCGTGGGCTCGG |
| SB710 | CAAGCAGAAGACGGCATACGAGATTACGAGCAGTCTCGTGGGCTCGG |
| SB711 | CAAGCAGAAGACGGCATACGAGATTCAGCGTTGTCTCGTGGGCTCGG |
| SB712 | CAAGCAGAAGACGGCATACGAGATTCGCTACGGTCTCGTGGGCTCGG |

**Table A3.** Primer array combinations for bacterial and fungal communities sequencing for 2^nd^ step PCR.

| **Array A** |  |  |  |  |  |  |  |  |  |  |  |  |
| --- | --- | --- | --- | --- | --- | --- | --- | --- | --- | --- | --- | --- |
|  | 1 | 2 | 3 | 4 | 5 | 6 | 7 | 8 | 9 | 10 | 11 | 12 |
| A | SA701 + SA501 | SA702 + SA501 | SA703 + SA501 | SA704 + SA501 | SA705 + SA501 | SA706 + SA501 | SA707 + SA501 | SA708 + SA501 | SA709 + SA501 | SA710 + SA501 | SA711 + SA501 | SA712 + SA501 |
| B | SA701 + SA502 | SA702 + SA502 | SA703 + SA502 | SA704 + SA502 | SA705 + SA502 | SA706 + SA502 | SA707 + SA502 | SA708 + SA502 | SA709 + SA502 | SA710 + SA502 | SA711 + SA502 | SA712 + SA502 |
| C | SA701 + SA503 | SA702 + SA503 | SA703 + SA503 | SA704 + SA503 | SA705 + SA503 | SA706 + SA503 | SA707 + SA503 | SA708 + SA503 | SA709 + SA503 | SA710 + SA503 | SA711 + SA503 | SA712 + SA503 |
| D | SA701 + SA504 | SA702 + SA504 | SA703 + SA504 | SA704 + SA504 | SA705 + SA504 | SA706 + SA504 | SA707 + SA504 | SA708 + SA504 | SA709 + SA504 | SA710 + SA504 | SA711 + SA504 | SA712 + SA504 |
| E | SA701 + SA505 | SA702 + SA505 | SA703 + SA505 | SA704 + SA505 | SA705 + SA505 | SA706 + SA505 | SA707 + SA505 | SA708 + SA505 | SA709 + SA505 | SA710 + SA505 | SA711 + SA505 | SA712 + SA505 |
| F | SA701 + SA506 | SA702 + SA506 | SA703 + SA506 | SA704 + SA506 | SA705 + SA506 | SA706 + SA506 | SA707 + SA506 | SA708 + SA506 | SA709 + SA506 | SA710 + SA506 | SA711 + SA506 | SA712 + SA506 |
| G | SA701 + SA507 | SA702 + SA507 | SA703 + SA507 | SA704 + SA507 | SA705 + SA507 | SA706 + SA507 | SA707 + SA507 | SA708 + SA507 | SA709 + SA507 | SA710 + SA507 | SA711 + SA507 | SA712 + SA507 |
| H | SA701 + SA508 | SA702 + SA508 | SA703 + SA508 | SA704 + SA508 | SA705 + SA508 | SA706 + SA508 | SA707 + SA508 | SA708 + SA508 | SA709 + SA508 | SA710 + SA508 | SA711 + SA508 | SA712 + SA508 |
|  |  |  |  |  |  |  |  |  |  |  |  |  |
| **Array B** |  |  |  |  |  |  |  |  |  |  |  |  |
|  | 1 | 2 | 3 | 4 | 5 | 6 | 7 | 8 | 9 | 10 | 11 | 12 |
| A | SA701 + SB501 | SA702 + SB501 | SA703 + SB501 | SA704 + SB501 | SA705 + SB501 | SA706 + SB501 | SA707 + SB501 | SA708 + SB501 | SA709 + SB501 | SA710 + SB501 | SA711 + SB501 | SA712 + SB501 |
| B | SA701 + SB502 | SA702 + SB502 | SA703 + SB502 | SA704 + SB502 | SA705 + SB502 | SA706 + SB502 | SA707 + SB502 | SA708 + SB502 | SA709 + SB502 | SA710 + SB502 | SA711 + SB502 | SA712 + SB502 |
| C | SA701 + SB503 | SA702 + SB503 | SA703 + SB503 | SA704 + SB503 | SA705 + SB503 | SA706 + SB503 | SA707 + SB503 | SA708 + SB503 | SA709 + SB503 | SA710 + SB503 | SA711 + SB503 | SA712 + SB503 |
| D | SA701 + SB504 | SA702 + SB504 | SA703 + SB504 | SA704 + SB504 | SA705 + SB504 | SA706 + SB504 | SA707 + SB504 | SA708 + SB504 | SA709 + SB504 | SA710 + SB504 | SA711 + SB504 | SA712 + SB504 |
| E | SA701 + SB505 | SA702 + SB505 | SA703 + SB505 | SA704 + SB505 | SA705 + SB505 | SA706 + SB505 | SA707 + SB505 | SA708 + SB505 | SA709 + SB505 | SA710 + SB505 | SA711 + SB505 | SA712 + SB505 |
| F | SA701 + SB506 | SA702 + SB506 | SA703 + SB506 | SA704 + SB506 | SA705 + SB506 | SA706 + SB506 | SA707 + SB506 | SA708 + SB506 | SA709 + SB506 | SA710 + SB506 | SA711 + SB506 | SA712 + SB506 |
| G | SA701 + SB507 | SA702 + SB507 | SA703 + SB507 | SA704 + SB507 | SA705 + SB507 | SA706 + SB507 | SA707 + SB507 | SA708 + SB507 | SA709 + SB507 | SA710 + SB507 | SA711 + SB507 | SA712 + SB507 |
| H | SA701 + SB508 | SA702 + SB508 | SA703 + SB508 | SA704 + SB508 | SA705 + SB508 | SA706 + SB508 | SA707 + SB508 | SA708 + SB508 | SA709 + SB508 | SA710 + SB508 | SA711 + SB508 | SA712 + SB508 |

**Table A3.** Primer array combinations for bacterial and fungal communities sequencing for 2^nd^ step PCR (cont.).

| **Array C** |  |  |  |  |  |  |  |  |  |  |  |  |
| --- | --- | --- | --- | --- | --- | --- | --- | --- | --- | --- | --- | --- |
|  | 1 | 2 | 3 | 4 | 5 | 6 | 7 | 8 | 9 | 10 | 11 | 12 |
| A | SB701 + SB501 | SB702 + SB501 | SB703 + SB501 | SB704 + SB501 | SB705 + SB501 | SB706 + SB501 | SB707 + SB501 | SB708 + SB501 | SB709 + SB501 | SB710 + SB501 | SB711 + SB501 | SB712 + SB501 |
| B | SB701 + SB502 | SB702 + SB502 | SB703 + SB502 | SB704 + SB502 | SB705 + SB502 | SB706 + SB502 | SB707 + SB502 | SB708 + SB502 | SB709 + SB502 | SB710 + SB502 | SB711 + SB502 | SB712 + SB502 |
| C | SB701 + SB503 | SB702 + SB503 | SB703 + SB503 | SB704 + SB503 | SB705 + SB503 | SB706 + SB503 | SB707 + SB503 | SB708 + SB503 | SB709 + SB503 | SB710 + SB503 | SB711 + SB503 | SB712 + SB503 |
| D | SB701 + SB504 | SB702 + SB504 | SB703 + SB504 | SB704 + SB504 | SB705 + SB504 | SB706 + SB504 | SB707 + SB504 | SB708 + SB504 | SB709 + SB504 | SB710 + SB504 | SB711 + SB504 | SB712 + SB504 |
| E | SB701 + SB505 | SB702 + SB505 | SB703 + SB505 | SB704 + SB505 | SB705 + SB505 | SB706 + SB505 | SB707 + SB505 | SB708 + SB505 | SB709 + SB505 | SB710 + SB505 | SB711 + SB505 | SB712 + SB505 |
| F | SB701 + SB506 | SB702 + SB506 | SB703 + SB506 | SB704 + SB506 | SB705 + SB506 | SB706 + SB506 | SB707 + SB506 | SB708 + SB506 | SB709 + SB506 | SB710 + SB506 | SB711 + SB506 | SB712 + SB506 |
| G | SB701 + SB507 | SB702 + SB507 | SB703 + SB507 | SB704 + SB507 | SB705 + SB507 | SB706 + SB507 | SB707 + SB507 | SB708 + SB507 | SB709 + SB507 | SB710 + SB507 | SB711 + SB507 | SB712 + SB507 |
| H | SB701 + SB508 | SB702 + SB508 | SB703 + SB508 | SB704 + SB508 | SB705 + SB508 | SB706 + SB508 | SB707 + SB508 | SB708 + SB508 | SB709 + SB508 | SB710 + SB508 | SB711 + SB508 | SB712 + SB508 |
|  |  |  |  |  |  |  |  |  |  |  |  |  |
| **Array D** |  |  |  |  |  |  |  |  |  |  |  |  |
|  | 1 | 2 | 3 | 4 | 5 | 6 | 7 | 8 | 9 | 10 | 11 | 12 |
| A | SB701 + SA501 | SB702 + SA501 | SB703 + SA501 | SB704 + SA501 | SB705 + SA501 | SB706 + SA501 | SB707 + SA501 | SB708 + SA501 | SB709 + SA501 | SB710 + SA501 | SB711 + SA501 | SB712 + SA501 |
| B | SB701 + SA502 | SB702 + SA502 | SB703 + SA502 | SB704 + SA502 | SB705 + SA502 | SB706 + SA502 | SB707 + SA502 | SB708 + SA502 | SB709 + SA502 | SB710 + SA502 | SB711 + SA502 | SB712 + SA502 |
| C | SB701 + SA503 | SB702 + SA503 | SB703 + SA503 | SB704 + SA503 | SB705 + SA503 | SB706 + SA503 | SB707 + SA503 | SB708 + SA503 | SB709 + SA503 | SB710 + SA503 | SB711 + SA503 | SB712 + SA503 |
| D | SB701 + SA504 | SB702 + SA504 | SB703 + SA504 | SB704 + SA504 | SB705 + SA504 | SB706 + SA504 | SB707 + SA504 | SB708 + SA504 | SB709 + SA504 | SB710 + SA504 | SB711 + SA504 | SB712 + SA504 |
| E | SB701 + SA505 | SB702 + SA505 | SB703 + SA505 | SB704 + SA505 | SB705 + SA505 | SB706 + SA505 | SB707 + SA505 | SB708 + SA505 | SB709 + SA505 | SB710 + SA505 | SB711 + SA505 | SB712 + SA505 |
| F | SB701 + SA506 | SB702 + SA506 | SB703 + SA506 | SB704 + SA506 | SB705 + SA506 | SB706 + SA506 | SB707 + SA506 | SB708 + SA506 | SB709 + SA506 | SB710 + SA506 | SB711 + SA506 | SB712 + SA506 |
| G | SB701 + SA507 | SB702 + SA507 | SB703 + SA507 | SB704 + SA507 | SB705 + SA507 | SB706 + SA507 | SB707 + SA507 | SB708 + SA507 | SB709 + SA507 | SB710 + SA507 | SB711 + SA507 | SB712 + SA507 |
| H | SB701 + SA508 | SB702 + SA508 | SB703 + SA508 | SB704 + SA508 | SB705 + SA508 | SB706 + SA508 | SB707 + SA508 | SB708 + SA508 | SB709 + SA508 | SB710 + SA508 | SB711 + SA508 | SB712 + SA508 |
